# Prevalence of Burnout Syndrome and Fear of COVID-19 among Adolescent University Students

**DOI:** 10.3390/children10020243

**Published:** 2023-01-30

**Authors:** Raimundo Aguayo-Estremera, Gustavo R. Cañadas, Luis Albendín-García, Elena Ortega-Campos, Tania Ariza, Carolina S. Monsalve-Reyes, Emilia Inmaculada De la Fuente-Solana

**Affiliations:** 1 Departamento de Psicobiología y Metodología de las Ciencias del Comportamiento, Facultad de Psicología, Universidad Complutense de Madrid, España, Campus de Somosaguas, Ctra. De Húmera, s/n, 28223 Pozuelo de Alarcón, Spain; 2 Department of Didactic of Mathematics, Faculty of Education Science, University of Granada, Campus de Cartuja s/n, 18071 Granada, Spain; 3 Casería de Montijo Health Center, Granada-Metropolitan Heath District, Andalusian Heath Service, Calle Virgen de la Consolación, 12, 18015 Granada, Spain; 4 Center for Health Research-UAL (CEINSA-UAL), Universidad de Almería, Carretera de Sacramento s/n, La Cañada de San Urbano, 04120 Almería, Spain; 5 Department of Educational Psychology and Psychobiology, Faculty of Education, Universidad Internacional de La Rioja (UNIR), Av. De la Paz, 137, 26006 Logroño, Spain; 6 Departamento de Ciencias Sociales, Universidad Católica de La Santísima Concepción, Concepción 4030000, Chile; 7 Brain, Mind and Behaviour Research Center (CIMCYC), University of Granada, 18071 Granada, Spain

**Keywords:** burnout syndrome, COVID-19, personality, prevalence, university students

## Abstract

This study aimed to estimate the prevalence of burnout syndrome in adolescents entering university studies, to detect differences in burnout levels, personality factors and fear of coronavirus in a pandemic context due to COVID-19. A cross-sectional predictive study was performed with a sample that comprised 134 individuals in their first year of a Psychology degree at Spanish universities. The Maslach Burnout Inventory Student Survey, the NEO Five-Factor Inventory and the Fear of COVID-19 Scale were applied. The prevalence of burnout is estimated according to three methods: Maslach and Jackson’s severity classification, Golembiewski’s phase model and Maslach et al.’s profile model. The estimates show significant differences. The results indicated that between 9 and 21% of students were at risk of developing burnout. On the other hand, students who reported having suffered psychological consequences of the pandemic showed greater emotional exhaustion, neuroticism and fear of COVID-19, and a lower level of personal accomplishment than those who did not suffer such consequences. Neuroticism was the only significant predictor for all burnout dimensions, and fear of COVID-19 did not contribute to any of them.

## 1. Introduction

Researchers have long been aware of the need to study burnout syndrome in students [1,2]. However, it was not until 2008 that the term school burnout was first used [3,4]. Burnout syndrome is a psychological problem that was originally associated with care workers, such as nurses and doctors, and which until recently was only studied with respect to workers in their employment environment. Research into burnout among students has been facilitated by the development of the Maslach Burnout Inventory—General Survey (MBI-GS) [5], which not only allowed the syndrome to be evaluated in all types of workers, but also offered the possibility of extending this study to students. This opportunity was taken by the authors of [6], who modified the MBI-GS items to be applied to students, as the Maslach Burnout Inventory—Student Survey (MBI-SS).

It might be said that schools, colleges and universities are workplaces for students. Although they are neither employed nor paid, from a psychological perspective the main tasks performed by students can be considered as work [4]. In the context of the job demands-resources theory [7], the parallel drawn between students and workers seems reasonable: students must travel regularly to class, where they spend a significant part of the day and must respond to the demands of a manager (the teacher) and fulfil standard tasks (exams, assignments, etc.), for which resources are provided. In some cases, indeed, task performance is accompanied by economic incentives such as grants or scholarships.

For these reasons, school burnout syndrome is defined using the same three-dimensional model originally proposed by the authors of [8] to characterize the syndrome in workers. In the new model, burnout is defined as a psychological problem derived from continual exposure to stressors related to school and studies, which is made up of three dimensions: emotional exhaustion, depersonalization and low personal accomplishment [4,8,9,10].

Emotional exhaustion refers to the feeling of tension experienced in the study environment, and in particular to the chronic fatigue that may result from academic overload. Depersonalization is expressed as indifference or alienation towards schoolwork in general, a loss of interest in one’s own academic work and a view of study as meaningless. Low personal achievement refers to the perception of decreased efficacy when studying and in the academic achievements achieved, and to a lack of value in the tasks performed and in school in general [6].

Student burnout can have serious consequences for the physical and psychological health of students [11], which may be psychosomatic (e.g., cardiovascular and gastrointestinal problems, lack of sleep, fatigue), emotional (e.g., depression, lack of self-esteem, demotivation) and/or behavioral (e.g., poor academic performance, alcohol and drug abuse, voluntary absences from classes, poor diet, absenteeism, dropping out).

The prevalence of burnout in students seems to be comparable to that observed in workers [12]. For example, rates of severe burnout ranging from 17% to 53% have been observed in university students [11]. In secondary school students, the corresponding figures are 10%–15% [4,13]. In general, studies of school burnout have either been conducted with high school students (aged under 18 years) or university students in general, with ages ranging from 17 to (in general) 24 years. Moreover, population samples of university students may include those taking master’s or doctoral degrees, as well as older adults and even elderly people. This type of composition would greatly and unreasonably increase the heterogeneity of the sample considered. To our knowledge, no previous study has been conducted to determine the prevalence of burnout syndrome among first-year university students, aged exclusively 17–19 years, with a high concentration of 18-year-old adolescents.

We believe it important to study burnout syndrome among a population of first-year students because these adolescents undergo a major change in life when they finish secondary education and start university. A university course can be very demanding, exposing students to a wide variety of stress-generating factors [14]. In addition to academic tasks, they may have to change their place of residence, learn university dynamics, live independently for the first time and develop new interpersonal relationships [15]. These changes are most dramatically felt by first-year undergraduates, and if they persist without there being adequate resources to address the problems that arise, the student may develop burnout syndrome.

In view of the scant body of research considering the impact of burnout on first-year university students, and the importance of this question, our aim is to achieve a better understanding of the effects of burnout syndrome among this population. Specifically, our primary goal is to determine the prevalence of burnout syndrome among first-year university students, taking into consideration the following classifications that have been proposed, according to the severity of the syndrome: (a) the proposal by the authors of [8], which classified those affected according to a low, medium or high degree of severity in each of the dimensions of the syndrome; (b) the proposal by the authors of [16], which grouped those evaluated into eight phases of severity, formed by combining high and low levels for each dimension of the syndrome; and (c) the proposal by the authors of [17], which uses five grouping profiles based on combinations of low, moderate and high levels in the dimensions of burnout. In the present study, the prevalence of burnout syndrome was estimated using all three proposals and the results were compared.

It seems plausible that the COVID-19 pandemic has worsened conditions in education centers. Specifically, the changes in living conditions provoked during the pandemic and the restrictions imposed may have altered students’ behaviors and emotions, their engagement with the learning process and, ultimately, their psychological well-being. University students experienced certain levels of burnout from the COVID-19 pandemic onwards [18,19], resulting in high levels of uncertainty about their employability and career planning [20], intention to drop out [21] and increased likelihood of anxiety or depression [18,19,22,23]. Since the onset of the COVID-19 pandemic, university students had to adapt to new ways of teaching that made their learning more difficult, and the presence of burnout was associated with a decreased ability to cope with academic challenges, thus affecting performance [24]. Perceived stress arising from the pandemic, the lack of social interactions and the shift from traditional to online teaching put pressure on students. When students suffer from burnout, they perceive themselves as incompetent which affects learning [25,26].

There is no consensus on when students are most vulnerable to experiencing academic burnout. One study highlighted that it is most prevalent during the second part of their university education, especially since the COVID-19 pandemic [27], although another claimed that there was no difference according to the year of study [19].

Fear of COVID-19 has been shown to directly and indirectly affect the intention to drop out of school and as a consequence of symptoms associated with academic burnout [28]. Students who perceived a greater impact of COVID-19 on their studies scored higher on academic burnout [29]. In view of these considerations, our study incorporates some objectives related to the pandemic and to the fear of contagion.

The second objective of this study was to determine whether there were differences in the dimensions of burnout, in the students’ personalities and in their fear of COVID-19 according to aspects related to the pandemic; specifically, if they had suffered from COVID-19, if a family member or friend had died due to COVID-19 or whether they had suffered psychological and/or physical consequences from the social situation caused by COVID-19. Our third and final objective was to analyze the predictive power of personality variables and fear of COVID-19 with respect to the scores obtained for the dimensions of burnout syndrome.

## 2. Materials and Methods

### 2.1. Participants

The study sample was composed of 134 adolescents at various Spanish universities who were studying the first year of an undergraduate psychology course. The selection process was based on non-probabilistic sampling. Of these participants, 84% were female and all were aged eighteen years.

To determine the simple size, a power analysis study was performed taking into account the work by Richard, Bond, and Stokes-Zoota (2003), in which they summarized 322 meta-analyses with more than 25,000 published studies in the field of personality and social psychology. They reported that the average published effect is *r* = 0.21. Assuming an effect size of 0.21, with a power level of 80% and a confidence level of 95%, the required sample size is around 130, which is similar to the number of participants recruited.

### 2.2. Procedure

A cross-sectional predictive design was used [30]. The data were collected from each of the universities using the same procedure in every case, during the first quarter of 2021. The data collection took place in the classrooms, during academic hours and with the approval of the teaching staff involved. All participants gave prior informed consent, and were assured confidentiality and anonymity at all times.

### 2.3. Instruments

An ad hoc sociodemographic questionnaire was applied, obtaining data such as the students’ age and sex, whether they had had COVID-19, whether someone they knew had suffered from it, or had died from it and whether they had experienced psychological or physical consequences related to the social situation caused by the pandemic. The following measurement instruments were administered.

The Maslach Burnout Inventory—Student Survey (MBI-SS), adapted for use with Spanish speakers [6]. The MBI-SS is composed of 15 items scored on a seven-point response scale, which measures the three dimensions of burnout syndrome according to the original proposal by the authors of [8], namely emotional exhaustion (the depletion of psychological and emotional resources), depersonalization (feelings of cynicism and detachment) and reduced personal accomplishment (feelings of non-efficacy and inadequate performance).

Four of the five dimensions of the Spanish version of the NEO Five-Factor Inventory (NEO-FFI) were administered [31], namely neuroticism, extroversion, agreeableness and consciousness. This instrument consists of 48 items scored using a Likert-type response format with 1–5 points. Each of the sub-scales (dimensions) is composed of 12 items.

Finally, the Fear of COVID-19 Scale (FCV-19S), as adapted for a Spanish student population [32], was used. This instrument consists of seven items scored on a five-point response scale.

### 2.4. Data Analysis

All statistical analyses were performed using R 4.2.1. (R Core Team, 2022), according to the following procedure. First, descriptive statistics were calculated for all the study variables. Second, the corresponding percentages were calculated to quantify the proportions of students with each level of severity of burnout syndrome, according to the classifications proposed in the literature. Third, Welch’s t test of hypotheses was performed [33] to characterize the differences in the three dimensions of burnout syndrome according to the variables related to COVID-19. The assumptions of normality and homoscedasticity were verified. The effect size used was Cohen’s d. In addition, χ2 tests were performed on these variables to study differences by sex. In this case, the effect size used was Cohen’s w. Fourth, correlations were determined to assess the degree of relationship between the continuous variables. Several backward, stepwise multiple linear-regression models were constructed to assess the predictive capacity of the variables of personality and fear of COVID-19 on the dimensions of burnout syndrome. The assumptions of linearity, normality, homoscedasticity and multicollinearity were all verified. Finally, the omega coefficient was determined to estimate the reliability of the scales and subscales [34].

## 3. Results

The descriptive statistics for all the study variables are shown in Table 1. In relation to reliability, in all the scales, the omega coefficients were above the usual recommended value of 0.70 [35]. Among the variables related to COVID-19, 50% of the participants had suffered from COVID-19, 92% knew someone close to them who had been infected and 19% had experienced the death of such a person. Moreover, 78% believed they had suffered psychological consequences due to the social situation caused by the pandemic and 62% had suffered physical consequences for this reason.

In the burnout syndrome severity classification proposed by the authors of [8], 21.6% of the participants had high levels of emotional exhaustion (EE), 21.1% had strong depersonalization (D) and 25.4% had decreased personal accomplishment (PA). Table 2 partially reproduces the burnout profiles obtained, according to the classification of the authors of [17]. Such reproduction does not require special permission (see https://s100.copyright.com/AppDispatchServlet?publisherName=ELS&contentID=S2213058615300188&orderBeanReset=true). The most prevalent profile was that of engaged (assigned to 38.3% of the students), followed by ineffective (30.1%).

Table 3 shows the severity of the burnout experienced by the students, according to the phase model devised by the authors of [16]. Combining the levels of each burnout dimension, eight phases of the severity of the syndrome were obtained for the students sample, according to Golembiewski model. According to the model, 29.3% of the students (low D and EE and high PA) are in phase 1, while 21.1% of the students are in phase 8 (high D and EE and low PA). Between phases 1 and 8, there are six phases of severity of the syndrome, combining the levels of dimensions D, PA and EE respectively (HHL, LLL, HLL, LHH, HHH and LLH) [16]. In the latter, 21.1% of students presented burnout syndrome (high EE and D and low PA), while according to the model to be found in [17], the corresponding value was 9%.

Hypothesis tests were performed to assess the differences in the levels of burnout syndrome according to the COVID-19-related variables considered (see Table 4). Statistically significant results were obtained for the psychological consequences derived from the pandemic; the students who reported these consequences presented more EE, N and FCV and less PA than those who did not. The magnitude of these differences ranged from low to moderate, except as regards neuroticism and fear of COVID-19, in which cases the differences were large. The tests also showed that the students who had suffered from COVID-19 had a significantly higher level of extraversion than those who had not. In addition, the students who had had physical consequences from the pandemic presented significantly higher levels of neuroticism than those who did not report these consequences. In this case, the effect sizes were low to moderate.

No statistically significant differences between men and women were recorded for any of the dimensions of the syndrome: thus, for EE, (t(29.54) = −1.67, *p* = 0.104); for D, (t(28.47) = −0.28, *p* = 0.791) and for PA, (t(30.45) = 1.37, *p* = 0.182). Similarly, there were no significant gender-related differences for the coronavirus-related variables: for students who had had COVID-19, (χ2(1) = 0.14, *p* = 0.706); for those who knew someone who had had the disease, (χ2(1) = 0.34, *p* = 0.555); for those who knew someone who had died from this cause, (χ2(1) = 0.13, *p* = 0.710); for those who presented psychological consequences, (χ2(1) = 2.07, *p* = 0.150) and for those who presented physiological consequences, (χ2(1) = 0.01, *p* = 0.995).

An analysis of the correlations between all study variables was also performed (see Table 1). Moderate, statistically significant correlations were recorded between the dimensions of the burnout syndrome measured with the MBI-SS. In this respect, the correlation between EE and D was positive, while those between PA and EE and between PA and D were negative. A small, but statistically significant, positive correlation was observed between fear of COVID-19 and D. Among the personality variables, the correlation with FCV was small and statistically significant for neuroticism, consciousness and agreeableness. Thus, students with higher scores for fear of COVID-19 tended to have higher ones, too, for neuroticism and consciousness, and lower ones for agreeableness. For EE, the correlation was statistically significant with neuroticism, extraversion and consciousness; for D and PA, the correlation was statistically significant with all the personality variables.

The predictive models were generated taking each of the three dimensions of the burnout syndrome as criteria, and the personality variables and fear of coronavirus as predictors. All three models were statistically significant, as follows: F(2, 123) = 28.19, *p* < 0.001, R2Adj. = 0.300 in EE; F(3, 122) = 7.31, *p* < 0.001, R2Adj. = 0.132 in D and F(3, 122) = 22.11, *p* < 0.001, R2Adj. = 0.336 in PA. As shown in Table 5, neuroticism was a statistically significant predictor in all three models. Among the other personality variables, extraversion and consciousness were significant in several models. Fear of COVID-19 only remained as a predictor for D, and even then was not statistically significant.

## 4. Discussion

School burnout syndrome is a psychological problem that arises from chronic exposure to academic stressors. This problem is serious and is suffered by a significant proportion of students, ranging from 9% to 53% according to different studies [4,11,13]. To our knowledge, no previous study has estimated the prevalence of the syndrome among 18-year-old university students. We believe such a study is necessary because it is precisely at this period of late adolescence when major changes must be faced (e.g., leaving home, making new interpersonal relationships, meeting new academic demands). If these circumstances are not addressed properly and resolved, the student may be at risk of suffering burnout. The present study aims to fill this gap in the literature, by estimating the prevalence of burnout syndrome in a sample of first-year students at Spanish universities.

Various methods for estimating this prevalence have been proposed, the best known being the approach used by the authors of [8] and the phase model provided by the authors of [16]. More recently, the authors of [17] proposed another method, based on analyzing latent profiles. The resulting model consisted of five profiles, derived from the scores obtained for the three dimensions of burnout (EE, D and PA). Our study is the first to report the prevalence of burnout comparing the results obtained by each of these three methods.

The results obtained in our analysis reveal discrepant results from the above methods. Although the procedure provided in [8] is not designed to produce a global estimate of prevalence, it showed that approximately 21% of the students in our sample were at risk. A very similar result was obtained with the severity phase method [16], by which 21.1% of the students were classified as phase eight, that is, at risk of suffering from burnout. However, with the new profile procedure [17], the proportion of students presenting burnout was only 9%. The latter method, therefore, seems to be more conservative than the earlier ones.

The measures adopted in response to the COVID-19 pandemic, including lockdowns, social isolation and (for students) virtual classes, were dramatic and unprecedented. In such circumstances, we believe it useful to consider whether infection with coronavirus or the experience of other unpleasant events related to the pandemic might have increased levels of burnout. Another question of interest concerns the fear of COVID-19 that adolescents may present, after these recent experiences, and its relationship with other psychological variables such as personality traits. Some studies conclude that burnout is present in university students, especially since the pandemic [18,19,27]. Our results show that levels of burnout were not increased by the fact of having experienced COVID-19, or if friends or relatives had had the disease or had died from it. We believe this absence of association is due to the fact that school burnout is a psychological problem affecting the academic context, and differs from other psychological characteristics such as depression and anxiety [36,37], which might be more directly affected by the circumstances mentioned [38]. In conclusion, general events such as suffering from COVID-19 seem to have only limited effect on school burnout. A striking result is that even levels of fear of COVID-19 were not exacerbated by having had the disease or by closeness to someone who had died from this cause. The fact that most of those who died from coronavirus were older adults or elderly (and that, in any case, the mortality figures were not high) might account for this result. In corroboration of this view, researchers have noted that adults and adolescents differ in their fear of COVID-19 [38]. Neither were significant effects of the same variables on personality traits detected, except for extraversion. In this respect, it seems that more extraverted students were infected with COVID-19 to a higher degree than those who were less extraverted, possibly due to a correlation between extraversion and risk perception [39], i.e., more extraverted students would perceive less risk of contagion and be more likely to engage in risky behavior.

The students who suffered psychological effects from the social situation caused by COVID-19 reported higher levels of emotional exhaustion, fear of coronavirus and neuroticism and lower levels of personal accomplishment than those who did not indicate such effects. From this, we conclude that the risk of suffering burnout syndrome forms part of the psychological consequences arising from the social changes caused by COVID-19. Neuroticism and fear of coronavirus were also more present in the students who reported suffering physical consequences. Hence, it seems that those with a higher level of neuroticism are at the same time more susceptible to developing problems with physical health. These results suggest that what actually increases the students’ fear of COVID-19 is not having developed it or having close acquaintances who have suffered from it, or even died from it, but rather their having developed psychological or physical problems derived from situations of social change.

An interesting result from our correlation analysis is that higher scores for fear of COVID-19 were positively associated with neuroticism and consciousness and inversely so with agreeableness. In this respect, the predictive models highlight the importance of personality traits in the development of burnout syndrome, thus corroborating previous research findings [40,41,42]. Neuroticism and extraversion explained 30% of the variability in the EE scores. In other words, the students who present higher levels of neuroticism and are less extraverted are more prone to this dimension of the burnout syndrome. In fact, neuroticism was the only significant predictor in all the regression models: on the one hand, together with consciousness and fear of coronavirus (although these two variables were not statistically significant) it accounted for 13.2% of the variance of depersonalization; and on the other hand, together with extraversion and consciousness, it accounted for 33.6% of the variability in personal accomplishment (with consciousness being the strongest predictor of the three). Notably, fear of COVID-19 did not substantially contribute to the variance explained for any dimension of the syndrome. This suggests that stable behavior patterns (or distal factors), such as personality traits, can sometimes be more determinant in the development of burnout than an emotional reaction to specific, demanding situations (proximal factors).

### 4.1. Limitations

This study presents some limitations. Firstly, the generalization of the results is limited to the selected Spanish universities. Although the sample size is adequate for the analyses performed, it would be desirable to carry out future research with students from other regions and countries to increase the generalizability of the results. Second, the design does not allow us to establish cause–effect relationships. In this sense, it would be desirable to carry out new studies with longitudinal designs, which are appropriate for this type of objectives. Third, the results of the present study are exploratory, given that the variables used in the predictive models were not based on any formal psychological theory, such as the job demands-resources theory [7]. Future studies could test confirmatory models based on the results of this study combining a well-defined theoretical framework. Finally, given the sample size for some of the COVID-19-related variables, it is possible that there are low magnitude differences that were not detected in this study. Future research with a larger sample size could further investigate this topic.

### 4.2. Implications

School burnout syndrome is a major problem for students. According to our findings, 9–21% of students are at risk of developing burnout. The presence of high levels of the burnout dimensions (EE, D and PA) often provokes serious consequences for physical and psychological health [11]. Some of these consequences are especially relevant in the educational context, for example, lack of sleep, fatigue, demotivation, poor academic performance, alcohol and drug abuse, class absenteeism and abandonment of studies. To address this possibility, university managers should provide a comprehensive service of attention for newly-arrived students, for example, providing them with detailed information about the facilities, the institution, the scholarships available, sports possibilities, opportunities for social interaction and leisure, etc. From the perspective of the job demands-resources theory [7], these actions can give students the necessary resources to face the demands of university entry, converting them into challenges that foster engagement, rather than crises provoking burnout. Finally, fear of COVID-19 seems to be triggered not by the contagion itself, affecting the students themselves or their friends and relatives, but by the psychological problems that arise from the resulting social situation. This finding should be borne in mind by those responsible for implementing policies to achieve social persuasion, and is a factor to consider with respect to future emergency situations and vaccination campaigns.

Furthermore, resilience and healthy coping skills have been associated with lower levels of academic burnout [23,43]. It could be useful to develop them in students by integrating them as an element of training in university degrees.

## Figures and Tables

**Table 1:** Descriptive statistics and correlations for the study variables (N = 134).

Variable	M	SD	1	2	3	4	5	6	7	8
1.EE	13.58	7.03	0.86							
2.D	6.20	5.50	0.63 ***	0.84						
3.PA	23.28	6.28	−0.39 ***	−0.36 ***	0.77					
4.N	39.85	9.03	0.53 ***	0.35 ***	−0.35 ***	0.89				
5.A	41.73	5.45	−0.10	−0.19 *	0.14	−0.24 **	0.71			
6.E	42.22	8.21	−0.34 ***	−0.18 *	0.30 ***	−0.28 **	0.11	0.88		
7.C	41.46	6.40	−0.24 **	−0.23 **	0.52 ***	−0.27 **	0.16	0.19 *	0.86	
8.FCV	14.72	4.90	015	0.20*	−0.05	0.28 **	−0.24 **	−0.06	0.15 **	0.88

EE = Emotional exhaustion; D = Depersonalization; PA = Personal accomplishment; N = Neuroticism; A = Agreeableness; E = Extraversion; C = Consciousness; FCV = Fear of COVID-19. The values on the diagonal are omega coefficients. * *p* < 0.05. ** *p* < 0.01. *** *p* < 0.001.

**Table 2:** Classification of the participants according to the profile model of Leiter & Maslach (2016) [17].

Profile	EE	D	PA	%	n
Engaged	L	L	H	38.3	51
Ineffective	L-M	L-M	L	30.1	40
Overextended	H	L-M	L-M	19.5	26
Disengaged	L-M	H	L-M	3.0	4
Burnout	H	H	L	9.0	12

EE = Emotional exhaustion; D = Depersonalization; PA = Personal accomplishment; L = Low; M = Moderate; H = High.

**Table 3:** Classification of the participants according to the phase model of Golembiewski et al (1986) [16].

Phase	1	2	3	4	5	6	7	8
	Low Burnout (Phases 1, 2, 3)			Moderate (Phases 4, 5)		High Burnout (Phases 6, 7, 8)		
% (n)	29.3 (39)	3.8 (5)	13.5 (18)	6.8 (9)	6.0 (8)	9.0 (12)	10.5 (14)	21.1 (28)

EE = Emotional exhaustion; D = Depersonalization; PA = Personal accomplishment; L = Low; M = Moderate; H = High.

**Table 4:** Hypothesis tests to analyze differences in burnout according to the coronavirus-related variables.

Variable	Outcome	M (SD) No	M (SD) Yes	t (df)	*p*	d
Had COVID19						
	EE D PA N A E C FCV	13.9 (7.48) 6.4 (5.66) 22.9 (6.34) 40.4 (8.65) 41.3 (5.9) 40.5 (7.19) 41.7 (6.31) 15.4 (5.43)	13.3 (6.6) 6.0 (5.38) 23.6 (6.25) 39.3 (9.42) 42.2 (4.99) 43.8 (8.81) 41.2 (6.53) 14.0 (4.23)	0.49 (130) 0.34 (130.8) −0.67 (132) 0.69 (127.9) −0.94 (123.4) −2.33 (125.1) 0.40 (128) 1.63 (124.6)	0.625 0.732 0.503 0.489 0.349 0.021 0.693 0.105	0.08 0.06 0.12 0.12 0.16 0.41 0.07 0.28
Friend/relative had COVID19						
	EE D PA N A E C FCV	13.5 (6.63) 6.2 (5.8) 23.1 (6.55) 39.6 (9.11) 41.9 (5.26) 42.4 (7.95) 41.1 (6.36) 14.8 (5.1)	13.9 (8.72) 6.2 (5.8) 24.0 (5.01) 40.8 (8.85) 41.0 (6.25) 41.7 (9.37) 42.9 (6.51) 14.3 (3.94)	−0.22 (30.7) −0.01 (34.5) −0.79 (44.9) −0.57 (37.1) 0.70 (32.5) 0.31 (32.8) −1.21 (33.7) 0.58 (44.5)	0.824 0.997 0.431 0.571 0.489 0.760 0.232 0.566	0.06 0.00 0.15 0.12 0.17 0.08 0.28 0.11
Psychological consequences						
	EE D PA N A E C FCV	10.3 (6.24) 4.9 (4.57) 25.3 (6.14) 34.0 (8.84) 42.4 (5.72) 42.4 (7.51) 42.2 (5.77) 11.5 (3.69)	14.5 (6.99) 6.7 (5.71) 22.7 (6.23) 41.5 (8.41) 41.5 (5.39) 42.2 (8.43) 41.2 (6.58) 15.7 (4.81)	−3.18 (51.9) −1.62 (58) 2.04 (47.6) −4.06 (43.6) 0.71 (43.2) 0.16 (47.6) 0.80 (50.9) −5.09 (60.3)	0.002 0.110 0.046 <0.001 0.483 0.875 0.428 <0.001	0.62 0.30 0.42 0.88 0.15 0.03 0.16 0.91
Physiological consequences						
	EE D PA N A E C FCV	12.5 (6.64) 5.8 (5.13) 23.7 (6.6) 37.6 (8.22) 41.8 (5.13) 42.7 (7.88) 41.4 (7.00) 13.7 (4.63)	14.2 (7.26) 6.5 (5.76) 23.0 (6.14) 41.3 (9.3) 41.7 (5.71) 42.1 (8.4) 41.3 (5.91) 15.5 (4.93)	−1.42 (113.4) −0.71 (112.9) 0.65 (100.3) −2.40 (111.3) 0.13 (112.3) 0.42 (106.9) 0.05 (91.5) −2.15 (111.2)	0.159 0.480 0.514 0.018 0.897 0.675 0.961 0.034	0.25 0.12 0.12 0.42 0.02 0.08 0.01 0.38

SD = Standard deviation; df = Degrees of freedom, EE = Emotional exhaustion; D = Depersonalization; PA = Personal accomplishment; N = Neuroticism; A = Agreeableness; E = Extraversion; C = Consciousness; FCV = Fear of COVID-19.

**Table 5:** Multiple regression models for each dimension of burnout syndrome.

Variable	B	SE	95% CI for B		*p*	β
			LL	UL		
Emotional exhaustion						
Intercept	7.28	4.179				
Neuroticism	0.36	0.060	0.24	0.47	<0.001	0.46
Extraversion	−0.18	0.066	−0.31	−0.05	0.006	−0.22
Depersonalization						
Intercept	4.49	4.97				
Neuroticism	0.15	0.062	0.03	0.27	0.17	0.26
Consciousness	−0.15	0.087	−0.32	0.02	0.79	−0.18
Fear of COVID-19	0.14	0.010	−0.01	0.34	0.160	0.13
Personal accomplishment						
Intercept	3.76	5.653				
Neuroticism	−0.12	0.058	−0.24	−0.01	0.37	−0.17
Extraversion	0.12	0.68	−0.02	0.25	0.82	0.15
Consciousness	0.46	0.077	0.31	0.61	<0.001	0.46

SE = Standard error; CI = Confidence interval; LL = Lower limit; UL = Upper limit.

## Data Availability

Not applicable.
